# Ultraspecific probes for high throughput HLA typing

**DOI:** 10.1186/1471-2164-10-85

**Published:** 2009-02-20

**Authors:** Chen Feng, Catherine Putonti, Meizhuo Zhang, Rick Eggers, Rahul Mitra, Mike Hogan, Krishna Jayaraman, Yuriy Fofanov

**Affiliations:** 1 Department of Computer Science, University of Houston, Houston, TX, USA; 2 Department of Computer Science, Loyola University Chicago, Chicago, IL, USA; 3 Department of Biology, Loyola University Chicago, Chicago, IL, USA; 4 Collaborative Center for Statistics in Science, Yale University, New Haven, CT, USA; 5 Genomics USA, Inverness, IL, USA; 6 Department of Biology and Biochemistry, University of Houston, Houston, TX, USA

## Abstract

**Background:**

The variations within an individual's HLA (Human Leukocyte Antigen) genes have been linked to many immunological events, e.g. susceptibility to disease, response to vaccines, and the success of blood, tissue, and organ transplants. Although the microarray format has the potential to achieve high-resolution typing, this has yet to be attained due to inefficiencies of current probe design strategies.

**Results:**

We present a novel three-step approach for the design of high-throughput microarray assays for HLA typing. This approach first selects sequences containing the SNPs present in all alleles of the locus of interest and next calculates the number of base changes necessary to convert a candidate probe sequences to the closest subsequence within the set of sequences that are likely to be present in the sample including the remainder of the human genome in order to identify those candidate probes which are "ultraspecific" for the allele of interest. Due to the high specificity of these sequences, it is possible that preliminary steps such as PCR amplification are no longer necessary. Lastly, the minimum number of these ultraspecific probes is selected such that the highest resolution typing can be achieved for the minimal cost of production. As an example, an array was designed and *in silico *results were obtained for typing of the HLA-B locus.

**Conclusion:**

The assay presented here provides a higher resolution than has previously been developed and includes more alleles than previously considered. Based upon the *in silico *and preliminary experimental results, we believe that the proposed approach can be readily applied to any highly polymorphic gene system.

## Background

The HLA (human leukocyte antigen) system, the group of genes in the human MHC (major histocompatibility complex) located on chromosome VI, encodes the cell-surface antigen-presenting proteins. HLA antigens are the major determinants used by the body's immune system for recognition and differentiation of self from non-self (foreign) substances. This system consists of numerous SNPs (single nucleotide polymorphisms) encoding more than 2,000 known alleles [1]. The allelic composition in the HLA loci, or the HLA type, varies significantly within the population. Research has identified a direct correlation between an individual's HLA type and his susceptibility to disease including Hodgkin's lymphoma [2], preeclampsia [3], diabetes [4], and Alzheimer's [5]. An individual's response to viral infection or drug treatment is also affected by their HLA type, e.g. variability in the course of human immunodeficiency virus type 1 (HIV-1) infection was shown to be partially the result of particular polymorphism in the HLA regions [6]. Furthermore, a single allele-level mismatch between a transplant donor and acceptor can dramatically reduce the survival rate of the recipient. The HLA types of the donors and acceptors have a significant effect on the occurrences of graft-versus-host disease (GVHD), engraftment failure, and graft-versus-leukemia effect [7,8].

Several different techniques have been explored for HLA typing. Historically, serological analysis of allele-specific sera was used to identify the structural differences on the surface of the HLA molecules due to the nucleotide present at the site of the polymorphism. This method, however, is limited by the availability of allele-specific sera and by its inability to discriminate between allelic subtypes within each serologic family [9]. An alternative means of typing is sequencing of the polymorphisms within the HLA loci [10,11]. For HLA class II genes, many of the polymorphisms are contained within exon 2 thus necessitating sequencing of only a few hundred bases. In contrast, the polymorphisms of class I genes require several exons to be sequenced which in turn introduces an increase in cost as well as the probability of sequencing errors. Furthermore, sequencing-based typing also suffers from inherent issues in resolving some sequencing miscalls. Despite this, sequencing has a substantially higher resolution than is possible with serological methods.

Numerous nucleic acid-based approaches have also been used for genotyping the HLA loci. One such approach, PCR-SSP (Polymerase Chain Reaction – Sequence Specific Primers), employs specific primers targeting a particular polymorphism such that the presence of the polymorphism results in amplification of the product [12]. Because the number of primers required depends upon the number of polymorphisms in the particular locus of interest, a large number of PCR reactions are typically needed to complete the HLA typing. PCR-SSOP (Sequence Specific Oligonucleotide Probes), another nucleic acid-based method, relies on PCR amplification of the locus of interest followed by probing with oligonucleotides designed to recognize specific SNPs [13]. The PCR step is essential for boosting the signal intensity and signal to noise ratio. Direct hybridization between SSOP and genomic DNA resulted in low signal intensity and the low signal to noise ratio, which may make the results difficult to interpret [14]. The resolution of both PCR-SSP and PCR-SSOP typing is low to medium as SNPs that are shared by several subtypes of the alleles often result in ambiguities. Because a simple PCR-SSP or PCR-SSOP hybridization might not result in the assignment of the HLA type, nucleic acid-based assays using the microarray technology have been tested for HLA typing within the past few years [15-21]. Microarrays are ideally suited for the high-throughput requirements by offering the convenience of miniaturization and the ability to perform thousands of hybridizations in a single experiment. This highly parallel nature of the microarrays and their unique format makes them ideally suited for field use as well. Microarray-based typing methods thus far discussed in the literature have thus far been able to identify only 6% to 33% of the allelic variations in the loci of interest.

Herein, a novel three-step approach for the development of high-throughput microarray assays for HLA typing is presented. First, candidate probe sequences (*n*-mers: subsequences of length *n*) are selected which contain the known SNPs present in the allelic sequences of the locus (or loci) of interest. Next, each candidate probes sequence is compared to the collection of sequences which are likely to be present in the sample (the "synthetic background"), including the remainder of the human genome and human-associated microbial genomic sequences. The distance, or the number of base changes that are necessary for a candidate probe sequence to be "converted" to the nearest sequence present in the synthetic background, is calculated for each probe. The candidate probes which are significantly distant from the background are less likely to misprime with, for instance, human genomic DNA. Such candidate probes are "ultraspecific" for identification of the allele even when foreign genomic material is present. It is possible that use of such ultraspecific probe sequences may eliminate the need of PCR amplification of the HLA region. Lastly, the smallest set of ultraspecific probes is selected such that the highest resolution typing can be achieved with a minimal cost in producing the assay. As an example, the proposed approach was applied to the most polymorphic region of the HLA system, the HLA-B locus.

## Results

The proposed method comprises of three steps.

1. Selection of candidate probes: Select all short subsequences (*n*-mers) of length 16 to 22 nucleotides (16 ≤ *n *≤ 22) containing each SNP in the allelic sequences producing the set of all candidate probes.

2. Identification of ultraspecific probes: Exclude from consideration all candidate probe sequences which are present in or can be converted to a subsequence in the synthetic background given any combination of two or more base change as such sequences are likely to have a high probability of mispairing with non-target DNA present in the sample. The results of this most computationally intensive step is the set of ultraspecific candidate probes or those sequences which are "distant" from the background collection.

3. Selection of optimal set of ultraspecific probes: Determine the set of ultraspecific probe sequences which requires the minimum number of members such that each known allele is expected to hybridize with a specific subset of probes.

For each of the 889 alleles within the HLA-B locus, the set of all 16- through 22-mer sequences were identified as candidate probe sequences. Thus, multiple *n*-mers containing the same SNP for the same allele were identified in which both the location of the SNP within the *n*-mer and the length of the *n*-mer vary. To utilize these probes as markers of the HLA-B specific polymorphisms, each of the candidate probes were compared to the background collection – the remainder of the human genome which includes the other highly similar HLA loci (e.g. HLA-A has 89% sequence similarity and HLA-C has 93% sequence similarity with HLA-B) and known polymorphisms outside of the HLA-B region. With regards to the ability to detect the set of alleles, all of the alleles contain 16- through 22-mers that are at least 1 change away. Table 1 lists the percentage of the alleles which can be detected by probes of a particular size and distance from the background; a complete listing of the number of candidate probes for each of the individual 889 alleles and distance values can be found in Additional File 1. To reduce the likelihood that probes would hybridize to non-target sequence by tolerating single base mispairing(s), we stipulate that ultraspecific *n*-mers must be 2 or more changes away from the background.

**Table 1:** Percentage of the 889 alleles that have at least one probe sequence for the lengths calculated for each of the distances from the background collection sequences.

	Candidate Probe Length						
Distance	16	17	18	19	20	21	22
1	100%	100%	100%	100%	100%	100%	100%

2	56.36%	99.55%	99.89%	100%	100%	100%	100%

3	0%	0%	0.11%	53.66%	93.36%	97.30%	99.89%

4+	0%	0%	0%	0%	0%	7.87%	13.61%

While these ultraspecific probe sequences, when incorporated in a microarray assay, are expected to be able to detect all of the 889 alleles considered, it is not guaranteed that a single assay could distinguish between different alleles. Thus, the set of ultraspecific probe sequences was analyzed using a greedy algorithm (see Methods) in order to generate the minimum set of ultraspecific probes required such that all of the alleles can be detected with the highest typing resolution possible. Based upon the results of our computations, a probe set including 115 ultraspecific probes was identified. Ultraspecific probes included in the set were selected based upon their ability to both detect the 889 alleles as well as their ability to provide resolution for distinguishing between alleles. Each allele is expected to hybridize with between two to 19 different probes in this set. While all ultraspecific probes are able to distinguish one to many different targets, not all members of the set provide the ability to indentify a particular allele (distinguishability). This set will produce distinctive hybridization patterns for 634 out of the 889 alleles such that 72.33% of the alleles can not only be detected but also definitively typed; the remaining 255 alleles, while detectable by this probe set, will not hybridize to any unique or unique combination of ultraspecific probe sequences.

**Figure 1:**
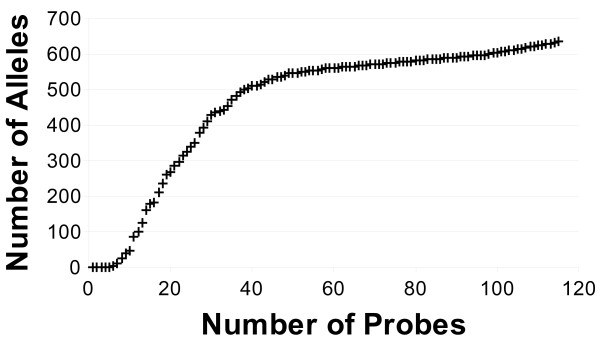
**Number alleles distinguished versus number of probes**.

**Figure 2:**
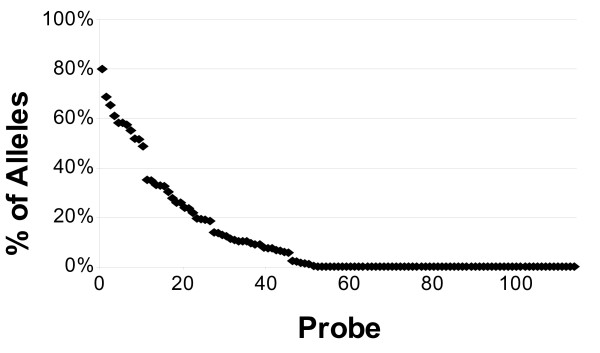
**Percentage of distinguishable alleles expected to hybridize with each of the 115 ultraspecific probes**.

As shown in Figure 1, the first five probes selected in the set are able to detect alleles but do not provide any distinction between the alleles detected; with the inclusion of the sixth probe, however, it is now possible to distinguish between the expected hybridization patterns of the B_1303 and B_560502 alleles from all other alleles considered. While the complete set of 115 ultraspecific probes can recognize 634 alleles, 40 of these ultraspecific probes are actually able to individually distinguish between 509 alleles (57.26%). Additional File 2 lists each of the ultraspecific probe sequences included in the set. The percentage of the 634 distinguishable alleles each of these probes is expected to hybridize with is shown in Figure 2. As is shown in this figure, nearly half (49.57%) of the probes included in the set are only expected to hybridize with a single allele thus generating a unique hybridization pattern such that the particular allele can be uniquely identified from all of the other alleles considered.

**Figure 3:**
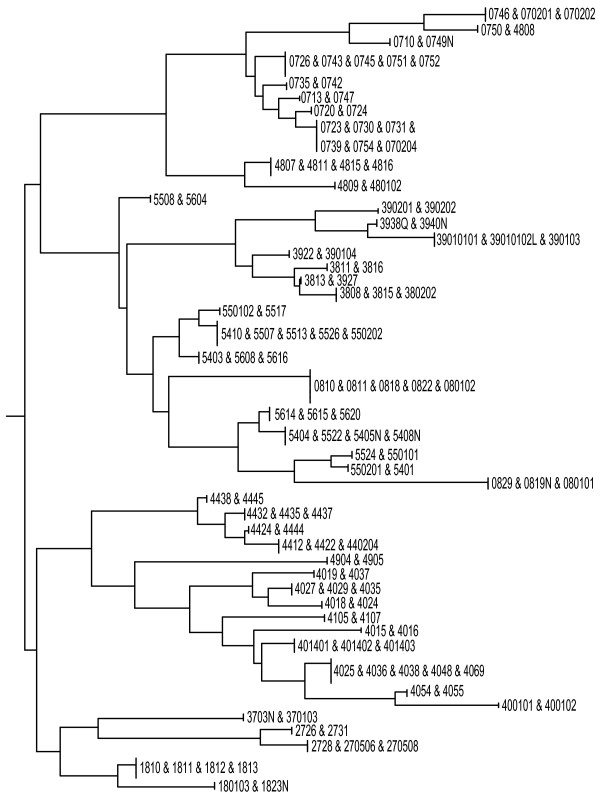
**Allele groupings based on the similarity of the observed hybridization patterns for the microarray of 115 ultraspecific probes**.

*In silico *assays for the 115 ultraspecific probe set and each of the alleles were conducted. As one would expect, it is the groups of highly similar alleles that the ultraspecific probe set is unable to distinguish between. In order to visualize the degree of similarity between the alleles based upon their expected hybridization patterns, we calculated the distance (D) between any two patterns as:

$$D=1-\frac{n_{12}}{\min(n_{1},n_{2})},$$

where *n*_1 _and *n*_2 _are the numbers of probes present in each of alleles being compared and *n*_12 _is number of probes present in both alleles simultaneously. By computing the distances between each pair of 889 patterns (the distance matrix) with PHYLIP's neighbor program [22], we were able to group alleles using the publicly available NJPlot software package [23] based on the distances between patterns observed on the microarray. The tree was generated for the set of alleles which cannot be individually typed. Figure 3 illustrates a subtree in this tree; the NEWICK file for the complete tree can be found in Additional File 3.

## Discussion and conclusion

The microarray of 115 probes represents what many researchers can produce in-house at low cost. While unable to uniquely identify every allele, the probe set presented here provides a higher resolution than has previously been developed and includes more alleles than previously considered. For the 255 alleles which cannot be distinguished, the nucleotide(s) present at the polymorphic positions defining the allele are included in candidate probe sequences that did not meet the ultraspecificity criteria. Incorporating probe sequences expected to hybridize within these polymorphic sites have a greater likelihood of interacting with regions outside of the HLA-B exons. As our goal here is to develop an assay such that amplification of the HLA loci exon prior to assaying is not necessary, such nonspecific probe sequences are excluded from the set. In the event that the assay generates a hybridization pattern common amongst several known alleles, further high resolution typing may be required. Furthermore, this assay lends itself towards exploratory typing. In the event that a novel hybridization pattern is observed, subsequent sequencing can be performed to determine the sequence of the new allele; when a known allele is encountered, however, the cost of this additional sequencing can be avoided. Based upon the results of *in silico *experiments as well as preliminary experimental results (Hogan *et al*., unpublished results), we believe that the proposed approach can be readily applied to the entire HLA system as well as other highly polymorphic gene systems. We are in the process of developing an "HLA-chip" to enable population-scale HLA typing in a portable, field-ready environment.

## Methods

### Sequence data

The catalogued set of resequenced alleles for class I HLA-B was retrieved from the IMGT/HLA Sequence Database (release 2.17 04/2007) [1]. The IMGT/HLA database provides multiple alignment for the allelic sequences with allele B*070201 as a reference. The aligned sequences are in a format such that the SNPs are clearly marked indicating the differences between the alleles. As specified by the IMGT database, this release contains 889 alleles within the HLA-B locus.

Sequences containing these SNPs from the allele exons were screened against the synthetic background of the set of genomic sequences likely to appear in the sample. This synthetic background includes the human genome (Build 36.1) with the exception of the HLA-B locus. The synthetic background was created by referencing the annotation (GenBank summary file) of the human genome build and removing the HLA-B locus' nucleotide sequence from the complete sequence of chromosome 6 . For simplicity, all *n*-mers containing unknown/unidentified characters were excluded from the calculations. The original and complementary strands were considered for both the HLA-B locus and the synthetic background collection.

### Candidate probe selection

All of the 16- through 22-mer sequences containing the SNPs present in each of the 889 allele sequences were identified. Based upon the results of calculations performed for the complete human genomic sequence for *n *<18, it was discovered that only when *n *≥ 16 that the human genome does not include a sequence within two changes of any selected *n*-mer [24]. Thus, candidate probe sequences less than 16 nucleotides long were not calculated as they would be removed from further consideration in the next step. Because the resolution of HLA typing using ultraspecific sequences longer than 22 did not provide any better resolution of typing (data not shown), computations for *n *greater than 22 are excluded from the results presented here.

### Identification of ultraspecific candidate probes

Using specialized algorithms and data structures [24], we rigorously computed what sequences are present in both the sequence of interest and present in the background as well as the number of base changes necessary to "convert" every *n*-mer contained within the sequence of interest to any *n*-mer present in the background collection. If any one combination is present in the background, for instance given two base changes, the *n*-mer is said to be two changes away from the nearest background sequence or "two away". By calculating the number of changes away each of the candidate probe sequences identified in the first step is from the background, it is possible to identify those candidate probes which are sufficiently "distant" from the background and thus have a reduced probability of hybridizing with non-target genomic material present in the sample.

### Probe set selection

To serotype HLA alleles, an assay must be able to not only detect the presence of the allele but also reliably distinguish between allelic variations. The issue of detection necessitates one to identify the set of ultraspecific candidate probes which are expected to hybridize with the particular allele. The ability to distinguish between alleles can be rather straightforward if each allele contains at least one candidate probe which is only expected to hybridize with that particular allele. Due to the high sequence similarity between the HLA-B alleles, however, the vast majority of the 889 alleles do not contain a unique candidate probe. The microarray platform provides an alternative means for approaching this challenge – rather than type a particular allele by a single unique probe, an allele can be identified by a unique set of probes such that an allele can be distinguished from another allele by its pattern of hybridization. A naïve solution is to incorporate all of the ultraspecific candidate probes. Due to the fact that the cost of microarray assays is directly proportionate to the number of probes included, it is ideal to design the assay with the minimal number of probes necessary in order to minimize cost and increase the ease in analyzing the assay results.

The problem of identifying the minimum number of probes to detect and distinguish a set of targets is an instance of the classical set cover problem, a well studied computational problem which is known to be NP Complete. Several algorithms have been designed to approximate the minimum probe set [24-29]. Herein we have developed another approach which has been integrated into our suite of algorithms and data structures. Our approach uses a greedy search strategy which is fast but does not guarantee to find the smallest probe set to detect and distinguish among all alleles. For each of the candidate probes, we calculate explicitly which targets the probe covers (is expected to hybridize with). We then cycle through a process of assessing the best candidate probe in terms of both the number of targets it will cover and its ability to improve the resolution in which individual targets can be identified. This selection process chooses probes that provide both coverage and distinguishability. In the event that the set of candidate probes remaining can provide only improved coverage or distinguishability, the probe set is assessed to see if the minimum coverage (number of times each target allele is expected to hybridize) specified by the user is met in which case candidate probes providing improved distinguishability are selected.

Once a probe is selected, it is included within the probe set and all targets that are covered by this probe and are distinguishable from all other targets are removed from consideration when assessing a future candidate probe's coverage of targets. Likewise, one can stipulate that each target is expected to hybridize with at least x different probes, in order to improve the reliability of the assay, such that when a probe is selected only targets meeting this threshold are removed from further consideration. This process of probe assessment and selection is repeated until either (a) all of the targets are expected to be detected by the probe set and distinguishable from either other based upon the subset of probes expected to hybridize with the individual target or (b) there are no more probes which can improve the detection and distinguishability of the set of targets. This approach was implemented a set of C++ data structures and efficient algorithms to select feasible sets among a large set of probes in a matter of seconds to minutes.

## Authors' contributions

CF participated in the code design and implementation, performing the computations, analysis of the results and drafting of the manuscript. CP participated in the design of the study, code development, analysis of the results and drafting of the manuscript. MZ participated in the design of the study and code development. RE, RM, MH, and KJ participated in the design of the study, experimental design critique, preliminary experimental design, and revision of the manuscript. YF conceived of the study and participated in its design and coordination. All authors read and approved the final manuscript.

## Supplementary Material

Additional file 1 **Additional table 1**. Complete listing of the number of candidate probes for each of the individual 889 alleles and distance values. Click here for file

Additional file 2 **Additional table 2.** The probe set including 115 ultraspecific probes for HLA-B typing. Click here for file

Additional file 3 **Additionalfile1**.txt. NEWICK file for the tree based upon the hybridization patterns for the undistinguishable alleles. Click here for file
